# Inflammation and TGF-β Signaling Differ between Abdominal Aneurysms and Occlusive Disease

**DOI:** 10.3390/jcdd6040038

**Published:** 2019-11-01

**Authors:** A. IJpma, L. te Riet, K. M. van de Luijtgaarden, P. M. van Heijningen, J. Burger, D. Majoor-Krakauer, E. V. Rouwet, J. Essers, H. J. M. Verhagen, I. van der Pluijm

**Affiliations:** 1 Department of Clinical Genetics, Erasmus University Medical Center, 3015 GD Rotterdam, The Netherlands; a.ijpma@erasmusmc.nl (A.I.); j.burger.1@erasmusmc.nl (J.B.); d.majoor-krakauer@erasmusmc.nl (D.M.-K.); 2 Department of Bioinformatics, Erasmus University Medical Center, 3015 GD Rotterdam, The Netherlands; 3 Department of Vascular Surgery, Erasmus University Medical Center, POB 2040 Rotterdam, The Netherlands; luukteriet@gmail.com (L.t.R.); k.vandeluijtgaarden@erasmusmc.nl (K.M.v.d.L.); e.rouwet@erasmusmc.nl (E.V.R.); j.essers@erasmusmc.nl (J.E.); 4 Department of Pharmacology, Erasmus University Medical Center, 3015 GD Rotterdam, The Netherlands; 5 Department of Molecular Genetics, Cancer Genomics, Erasmus University Medical Center Rotterdam, 3015 GD Rotterdam, The Netherlands; paula_van_heijningen@outlook.com; 6 Department of Radiation Oncology Erasmus University Medical Center, 3000 CA Rotterdam, The Netherlands

**Keywords:** abdominal aneurysm, occlusive disease, gene expression profiling, inflammation, TGF-β signaling

## Abstract

Abdominal aortic aneurysms (AAA), are usually asymptomatic until rupture causes fatal bleeding, posing a major vascular health problem. AAAs are associated with advanced age, male gender, and cardiovascular risk factors (e.g. hypertension and smoking). Strikingly, AAA and AOD (arterial occlusive disease) patients have a similar atherosclerotic burden, yet develop either arterial dilatation or occlusion, respectively. The molecular mechanisms underlying this diversion are yet unknown. As this knowledge could improve AAA treatment strategies, we aimed to identify genes and signaling pathways involved. We compared RNA expression profiles of abdominal aortic AAA and AOD patient samples. Based on differential gene expression profiles, we selected a gene set that could serve as blood biomarker or as pharmacological intervention target for AAA. In this AAA gene list we identified previously AAA-associated genes COL11A1, ADIPOQ, and LPL, thus validating our approach as well as novel genes; CXCL13, SLC7A5, FDC-SP not previously linked to aneurysmal disease. Pathway analysis revealed overrepresentation of significantly altered immune-related pathways between AAA and AOD. Additionally, we found bone morphogenetic protein (BMP) signaling inhibition simultaneous with activation of transforming growth factor β (TGF-β) signaling associated with AAA. Concluding our gene expression profiling approach identifies novel genes and an interplay between BMP and TGF-β signaling regulation specifically for AAA.

## 1. Introduction

Aneurysms are arterial dilatations with a diameter increase of more than 50% compared to normal. Aortic aneurysms can arise at different locations, and are commonly divided into thoracic and abdominal aortic aneurysms (TAA and AAA). Both aneurysm types are associated with age, smoking, hypertension, male gender, and atherosclerotic burden, as well as with environmental and familial components. Around 5% of TAAs are present in a syndromic form for which several genes have been identified so far [1,2].

Genes that have directly been linked to syndromic forms of TAA encode for TGF-β components, cytoskeleton proteins, or extracellular matrix (ECM) proteins. Well-known examples are Marfan syndrome (MFS) with a mutation in the extracellular matrix protein Fibrillin-1, Loeys-Dietz syndrome (LDS) with mutations in genes including the TGF-β-receptors 1 and 2, and SMAD3 [3,4,5]. The histology of aortic aneurysm sections of these patients usually shows abnormalities in the extracellular matrix (ECM), loss of smooth muscle cells, and disorganization of elastin and collagen structure [6,7]. Furthermore, TGF-β-signaling is increased in TAAs of patients and mice, and high serum TGF-β levels correlated directly with aortic root dilation [8,9,10,11]. It is unclear if genetic factors affected in TAA also play a role in AAA, though a recent study identified overlapping genetic defects between AAA and familial TAA [12]. The prevalence of AAA is ~6–9% among men older than 65 years of age [13,14,15] and is usually higher than for TAA with variations between populations globally [16]. Yet, in contrast to TAA, AAA causative genes have not been identified yet [17].

In men over 65 years of age, 48% have atherosclerosis in the aorta, of which 9–16% will also develop an aortic aneurysm [18,19]. Atherosclerosis is quite common in developed countries and contributes to major morbidity and mortality. Contributing factors such as dyslipidemia and hypertension will result in the manifestation of plaque development, vascular smooth muscle cell (VSMC) proliferation, and extracellular matrix modulation, eventually resulting into obstruction of the blood vessel as seen in AOD. Patients with abdominal aneurysms, with often similar atherosclerotic burden as AOD, show another form of extracellular matrix modulation and a different role of VSMCs [1].

Thus, identifying the underlying signaling pathways and genes will be a first step towards understanding how these two diseases differentiate despite their common risk factors. In this study we therefore investigated the transcriptional profile that differentiates AAA from AOD.

## 2. Materials and Methods

### 2.1. Tissue Analysis

#### 2.1.1. Patient Cohort Tissue Collection

Aortic tissue was derived from patients undergoing elective open surgical reconstruction of the infrarenal abdominal aorta for either abdominal aortic aneurysm (AAA) or aorto-iliac occlusive disease (AOD) in the Erasmus University Medical Center between 2008 and 2012. The study complies with the Helsinki declaration on research ethics. Aortic biopsies were obtained by protocol approved by the institutional Medical Ethics Committee (MEC-2012-387, MEC-2013-265, MEC-2014-057). Participants provided written informed consent.

In this study, the gene expression profiles of AAA patients were compared to the gene expression profiles of AOD patients with the goal of identifying the specific molecular changes that underlie the widening of the aorta as seen in the AAA patients. We chose this approach as, interestingly, both groups share the same risk factors and atherosclerotic burden, but in the AAA patient group the pathology is a widening of the aorta as opposed to occlusion in the AOD patient group.

#### 2.1.2. Aortic Biopsies

Full thickness aortic tissue samples for RNA expression profiling in AAA patients were collected from the infrarenal anterior aneurysm wall in AAA patients. Full thickness aortic tissue samples in AOD patients were obtained from the infrarenal anterior aortic wall at the site of the proximal anastomosis of the prosthetic graft. Tissue samples were rinsed in PBS and snap frozen in liquid nitrogen directly after harvesting and stored at −80 °C until RNA isolation.

#### 2.1.3. RNA Isolation and Microarray Hybridization

Total RNA including miRNAs were isolated using the miRNeasy Mini Kit (Qiagen, Hilden, Germany). Tissues were disrupted with a 5 mm steal bead by a disruption program of two times three min 20 Hz in the TissueLyser II (Qiagen, Hilden, Germany). RNA quality was checked with the Bioanalyzer 2100 (Agilent Technologies, Santa Clara, CA, USA). Samples with a high-quality RNA Integrity number (RIN) and with a 28S/18S ratio of >0.9 were used for hybridization. Microarray hybridization and scanning were performed at Skyline Diagnostics (Skyline Diagnostics, Rotterdam, The Netherlands). In short, 625 ng RNA was processed to generate cRNA. Fragmented and biotinylated cRNA was subsequently hybridized on Affymetrix U133 plus 2.0 microarrays (Affymetrix Inc., Santa Clara, CA, USA) and these microarrays were scanned with an Affymetrix Genechip System 3000Dx v.2 microarray scanner (Affymetrix Inc., Santa Clara, CA, USA).

#### 2.1.4. RNA Expression Analysis

The CEL files generated by the Affymetrix Genechip System 3000Dx v.2 microarray scanner were subsequently imported into Partek Genomics Suite version 6.4 (Partek Inc., St. Louis, MO, USA). Quantile normalization and background correction was applied to the raw intensity values of all samples via GC Robust Multichip Analysis. As the data was processed in three hybridization batches, hybridization batch effect correction was applied. To visualize the correlation between the samples, principal component analysis and unsupervised hierarchical clustering were used. For the comparison of AAA with AOD samples, two-sample *t*-test statistics were applied to calculate the fold changes with associated *p*-values.

#### 2.1.5. Microarray Data Processing

During data processing within Partek Genomics Suite 6.4, all microarray CEL files were assessed for passing of quality control (QC) thresholds. The analysis was started with 14 AAA samples and seven AOD samples. One AAA sample failed QC due to bad hybridization and this sample was removed from the analysis. During unsupervised clustering, all AOD samples grouped together and all AAA samples grouped together, except for one which grouped together with the AOD samples. Since all other AAA and AOD samples clustered into two distinct groups, and we could not rule out misidentification for this one AAA sample, it was removed from further analysis.

#### 2.1.6. Ingenuity Pathway Analysis

For the comparison between the AAA and AOD sample groups, the normalized expression values of 22,797 genes were uploaded into Qiagen Ingenuity Pathway Analysis (IPA) (Qiagen, Redwood City, CA, USA) and an IPA core analysis was performed on 1047 significantly differentially expressed genes (–2 <= FC => 2 and *p*-value ≤ 0.05). During upload of the data into IPA, the probe level data was mapped to the gene level and averaged based on the median fold change values. For the upstream analysis the z-score significance thresholds were set to –2.0 =< *z*-score >= 2.0 and *p*-value ≤ 0.01. As part of the core analysis, the functions, pathways, and upstream regulators were analyzed. In addition, information about molecule type and localization was derived from IPA.

#### 2.1.7. qPCR Analysis

Expression data of COL11A, Adiponectin, CXCL13, SLC7A5, and FDC-SP were analyzed in diseased aortic tissue (Table 1). Total RNA was reverse transcribed using iScript cDNA Synthesis Kit (Bio-Rad, Veenendaal, The Netherlands). cDNA samples were subjected to 40 cycles real-time PCR analysis using SYBR Green qPCR Master Mix 2x (Bio-Rad, Veenendaal, The Netherlands) and primers.

### 2.2. Clinical Study Population Analysis

#### 2.2.1. Patient Cohort Study Population

The study population consisted of a cohort of 1393 vascular surgery patients consecutively operated at the Erasmus Medical Center in Rotterdam between 2002 and 2011, as previously described [20]. Patients undergoing elective open or endovascular aortic aneurysm repair, peripheral arterial disease, or carotid artery disease, were included in the study. Patients were classified as aneurysmal disease (AA) or arterial occlusive disease (peripheral arterial disease or carotid artery disease). The study complies with the Helsinki declaration on research ethics and was approved by the institutional Review Board of the Erasmus Medical Center (MEC 2011-510). Clinical characteristics of this patient population were previously described [20].

#### 2.2.2. Lipoprotein and Inflammatory Parameters

Serum levels of triglycerides, high-density lipoprotein, low-density lipoprotein, and high-sensitive C-reactive protein (hs-CRP) were determined prior to surgery as described [21]. Patients with an hs-CRP higher than 10 mmol/L were excluded from analysis due to the chance of active inflammation status [21].

#### 2.2.3. Statistical Analysis

Dichotomous data are presented as numbers and percentages. Continuous variables are presented as mean ± standard deviation or median and interquartile range (IQR) when not normally distributed. Categorical data were analyzed with Fisher’s exact test or chi-square test and continuous variables with *t*-test, ANOVA, or Kruskal-Wallis test. For all tests, a *p*-value <0.05 (two-sided) was considered significant. Analyses were performed using Graphpad Software (Graphpad Software Inc., La Jolla, CA, USA) or SPSS statistics (version 21.0; IBM Inc., Chicago, IL, USA).

## 3. Results

### 3.1. AAA and AOD Patient and Sample Characteristics

In this study, we compared the RNA expression profiles of AAA samples to those of AOD samples. With this approach we excluded gene expression changes that result from shared risk factors as AOD samples serve as best-match control samples to identify genes and molecular pathways specifically related to AAA disease. The study included 12 AAA samples and 7 AOD samples and patient characteristics for the artic tissue used in this study are depicted in Table 2. The baseline characteristics showed a difference in age and gender, though as expected, no significant differences were found in cardiovascular risk factors such as diabetes mellitus, ischemic heart disease, renal insufficiency, hypertension, dyslipidemia, and smoking status.

### 3.2. Non-Supervised Hierarchical Clustering and Principal Component Analysis

**Figure 1:**
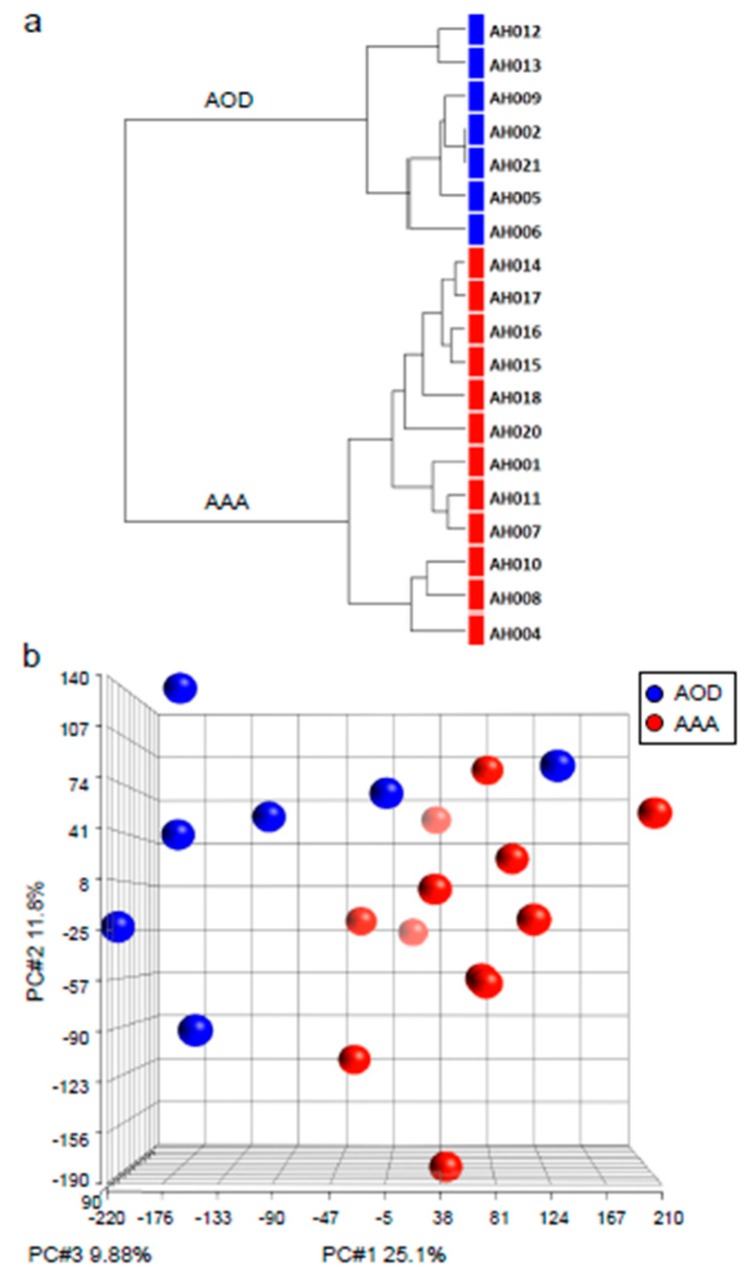
(**a**) Hierarchical clustering dendrogram of AAA (adominal aortic aneurysm) and AOD (arterial occlusive disease) samples. (**b**) Principal component analysis lot of AAA and AOD samples. In red the AAA patient samples, in blue the AOD patient samples. On the x, y, and z axis: PC#1 25.1%, PC#2 11.8%, PC#3 9.88%, respectively.

Non-supervised hierarchical clustering was performed on the genome wide microarray gene expression data of the 19 samples (Figure 1A). This analysis showed a clear separation of the AAA and AOD groups, and thus can be considered a validation of clear microarray gene expression differences between the two groups. In addition, principal component analysis (PCA) was performed on the samples and again a clear separation of the AAA and AOD samples was observed (Figure 1B).

### 3.3. Gender Difference Exclusion

Age and gender are important risk factors for AAA. In our dataset, 11 of the 12 AAA patients were male and 5 out of 7 AOD patients were female. Due to the overlap of gender with disease phenotype, our analysis could also potentially identify differences between males and females. To identify genes that are differentially expressed between male and female aortic samples we obtained microarray expression data from another study investigating AAA [22]. We downloaded the expression data from GEO (GSE 7084) and performed a two-sample *t*-test on the male and female sample groups within the control group only. This dataset consisted of an Affymetrix array-based analysis and an Illumina array-based analysis (for both array-based analysis: Two females versus four males). We identified genes as significantly differentially expressed in the Affymetrix analysis with *p*-value < 0.05 and FC cut off of > 3.5 whereas in the Illumina analysis we applied *p*-value < 0.05 and FC > 2.5 cut-offs. With these platform-specific stringent settings we identified 137 gender specific genes. As in the present study we were specifically interested in the genes that differentiate aneurysmal disease from occlusive disease irrespective of gender, these 137 gender specific genes were removed from our AAA versus AOD analysis. In Supplemental Table S1 we show a top selection of upregulated genes with 10 marked as gender specific (see M symbol in column). In addition, we performed an IPA core analysis on the dataset with and without the gender specific genes (1077 and 1047 ready molecules, respectively). Both analyses showed very similar results regarding functions, pathways, and upstream regulators, suggesting that the differences between AAA and AOD state are the predominant state difference in this dataset (data not shown). We next continued our AAA specific gene selection with all marked ‘gender-specific’ genes excluded.

### 3.4. Selection of Genes

**Figure 2:**
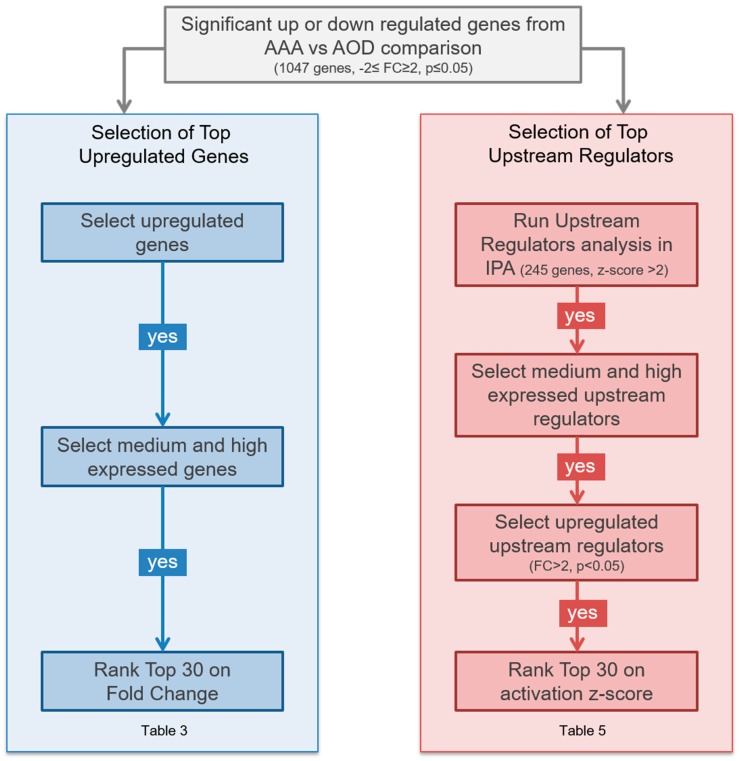
Selection procedure flowchart of the top upregulated genes (left) and top upstream regulators (right).

We applied a prioritization workflow to the set of differentially expressed genes, thus generating a list of top upregulated and key regulator genes based on their level of expression and their possible relevance for aneurysmal disease (AAA gene list). The two parts of the IPA core analysis that contributed to this prioritization workflow (Figure 2) are the list of significantly upregulated genes (part 1 of AAA gene list, Table 3) scored by highest fold change and *p*-value, together with the list of significant upstream regulators (part 2 of AAA gene list, Table 3, see below for further explanation). Upstream regulators are not necessarily differentially regulated themselves, but are identified based on the prediction that they regulate a significant set of genes present within the gene expression dataset being analyzed. The final AAA gene list consists of 60 genes; 30 based on selection of the most significantly upregulated genes (left selection procedure in Figure 2) and 30 genes based on the most significant upstream regulators (right selection procedure in Figure 2).

We took the following steps to prioritize the 30 most significantly upregulated genes: (1) The normalized raw expression values were divided into three categories: Low (<80), Medium (80–800) and High (>800). Only genes with High or Medium expression levels were considered as we reasoned it will be technically difficult to detect a gene with low expression values. (2) Only genes that showed an increase in expression levels in the AAA samples relative to the AOD samples were selected since detection of increased expression (presence) is more robust than detection of decreased expression (absence). (3) We included the protein localization of the expressed mRNA, as we reasoned that this would indicate the possibility to detect a potential marker in blood. (4) All genes that were also present in our in-house developed vascular gene set, were marked. The vascular gene set is a list of 4209 genes with relevance to vascular tissue development, maintenance and disease, including aortic aneurysms, that are selected based on HGMD and OMIM information, GO terms, KEGG pathways, Ingenuity IPA pathways, GWAS studies and literature (Supplemental Table S2). (5) As a last step, we also marked the genes that were identified as significant upstream regulators. Here, we reasoned that prioritization via two independent analyses gives increased overall confidence in the proper selection. This prioritized list was rank ordered based on the FC values.

### 3.5. Selection Procedure of Top Upregulated Genes Reveals ‘Known’ and ‘Novel’ Candidate Marker Genes’ for AAA

In our differential expression analysis, we identified both up and downregulated genes. However, for the analysis of dysregulated genes between the AAA and AOD patient groups we focused on genes upregulated in AAA with the aim of potentially identifying genes that could serve as biomarkers or pharmacological intervention targets. The top 30 upregulated genes (AAA gene list, part 1) were selected based on fold change and *p*-value and annotated with location and molecule type information as described above Table 3. In addition, we checked the presence of the top upregulated genes in our vascular gene set, which is an enriched gene set consisting of genes expressed in vascular tissues and/or having a role in vascular related pathways and functions. A literature search of the 10 most upregulated genes was performed where we screened for relevance in AAA or atherosclerotic disease (Table 4). Collagen-alpha1(XI) (COL11A1) appears highly relevant for AAA based on its location in the ECM and its previous association with aneurysmal disease [23]. Moreover, Adiponectin (ADIPOQ) seems relevant, as ADIPOQ is elevated in Kawasaki patients (aneurysms in coronary artery) [24]. Both associations show that our filtering is able to identify potential or known AAA-relevant genes. Furthermore, many highly upregulated genes are associated with the immune system, for instance CXC motif chemokine 13 (CXCL13), follicular dendritic cell secreted protein (FDC-SP), POU domain class 2-associating factor 1 (POU2AF1), membrane-spanning 4A (MS4A1 or CD20), and marginal zone B and B1 cell-specific protein (MZB1). Upregulation of these genes identifies the prominence of inflammation genes in AAA. Although the involvement of inflammation in both AAA and AOD is clear, in our literature research many of these identified genes showed no direct link with aneurysmal disease, therefore these genes could be novel.

**Figure 3:**
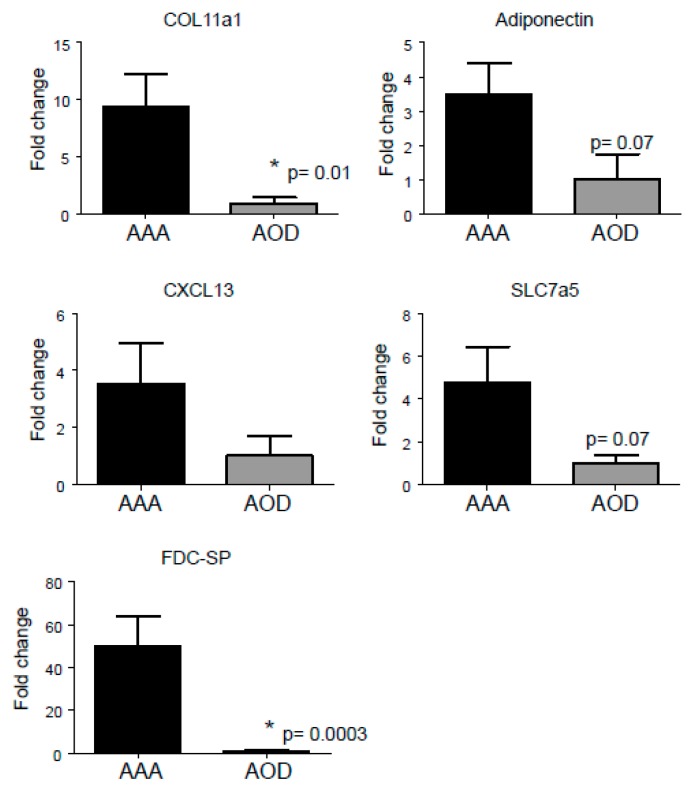
qPCR analysis of significantly regulated genes from the top 10 selection (Table 4), verified by qPCR. Plotted are the fold changes of COL11A1, ADIPOQ, CXCL13, SLC7a5, and FDCSP gene (AAA versus AOD *n* = 5). * *p* < 0.05 versus AAA.

A subset of five potential marker genes were selected from Table 4 to be verified by qPCR, as an additional check for the micro-array results. Selection criteria were increased fold change, extracellular location and an association with aneurysmal disease, resulting in selection of CXCL13, COL11A,1 and ADIPOQ. Additionally, FDC-SP and solute carrier family 7 member 5 (SLC7A5) were selected as they had not previously been associated with aneurysmal disease. qPCR data shows that COL11A1, ADIPOQ, CXCL13, SLC7A5, and FDC-SP are upregulated in AAA compared to AOD (Figure 3), which corresponds to the micro-array data, although only COL11A1 and FDC-SP were significantly upregulated. The other genes were upregulated, but without a significant *p*-value, which might be due to the small number of samples available for this analysis.

### 3.6. Selection of Top Upstream Regulators Indicating Potential Key Regulators in AAA

An additional selection procedure to identify novel genes in AAA was performed by using the differentially expressed gene data to identify top upstream regulators (Figure 2, right). The upstream regulator analysis is based on the idea that the activation state of a known upstream regulator can be determined by assessing the expression fold changes of all of its downstream targets and then using a z-score based algorithm to test if there is a good correlation between the hypothetical regulatory state of the upstream regulator and the regulatory state of all of its known downstream targets. The data was prioritized by highest upstream regulator *z*-score (*z*-score ≥ 2), with a minimal *p*-value of 0.01, and a FC ≥ 2, resulting in a gene list selected on the basis of upstream regulators. In addition, genes that were present in the vascular gene set were marked. Next, only upstream regulators that were identified as being significantly upregulated at the mRNA level in our dataset, with fold change ≥ 2 were selected. Here, we reasoned that prioritization via two independent analysis gives increased overall confidence in the proper selection. This prioritized list was rank ordered based on the z-score values. In Table 5 we show the top 30 upstream regulators (AAA gene list, part 2), ranked by z-score, together with their respective fold changes in the gene expression dataset. This list of genes will be used to indicate novel markers and key regulators involved in AAA disease, specifically those upstream regulators that also show a significant change at the transcriptional level (depicted with FC, fold change in Table 5).

### 3.7. Pathway Selection by Ingenuity Pathway Analysis Identifies Distinct Inflammatory Pathways in Aneurysmal and Arterial Occlusive Disease

**Figure 4:**
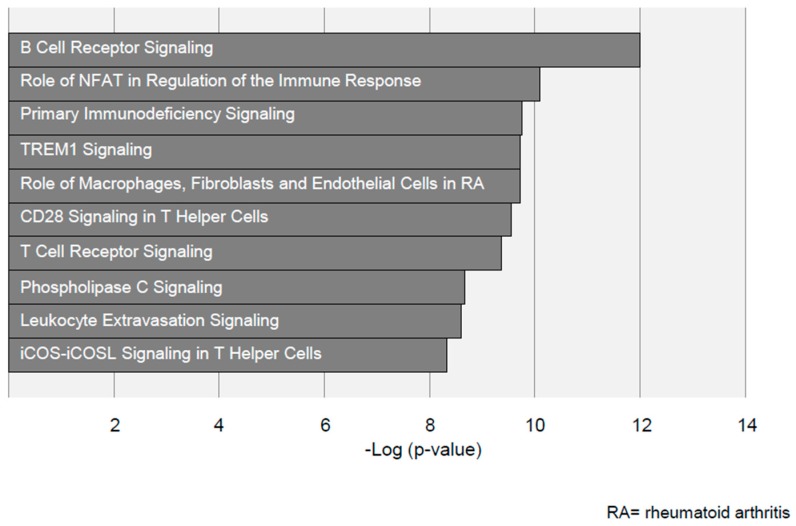
**List of the top** 10 significantly upregulated pathways in AAA disease identified with Ingenuity Pathway Analysis (IPA). The –log(*p*) value depicted on the x-axis represents significance of the depicted pathways.

Figure 4 shows a top 10 IPA list of pathways which are significantly altered in AAA disease. These top 10 pathways are all of an inflammatory nature, that could indicate the immune system as a differential component between AAA and AOD.

We therefore next re-examined the clinical characteristics from medical records of a large group of vascular surgery patients for indications of inflammation changes as previously described [20]. This population consisted of 1393 patients and included 614 patients (44%) treated for aortic aneurysms and 779 patients (56%) for arterial occlusive disease. Patients with occlusive disease included patients with peripheral arterial disease (*n* = 491) or carotid artery disease (*n* = 288). Endovascular procedures were performed in 598 patients (43%). The mean age of the population was 68 ± 10 years and the majority of patients were men (75%). Patient and baseline characteristics are described in [20]. In this patient cohort, a significant difference was observed between aneurysmal and occlusive disease patients in age (71 versus 66 years, respectively) and male gender (86% versus 67%, respectively), as was likewise present in our smaller patient group used for gene expression profiling, showing the representative nature of this patient cohort for the general population, and the samples used in this study. Previously, Ramnath et al. showed that the inflammatory marker high-sensitivity C-reactive protein (hs-CRP) was higher in AA patients than in AOD patients (5.9 versus 4.8 mg/L). We now re-examined these data by excluding hs-CRP values > 10 mmol/L (indicating active inflammation [21]). In Table 6 it is shown that the inflammatory marker hs-CRP was still slightly, though significantly higher in patients with AAA compared to occlusive disease (4.00 versus 3.00 mg/L) [21].

### 3.8. The TGF-β Pathway Is Significantly Regulated at Both Gene and Upstream Regulator Level

**Figure 5:**
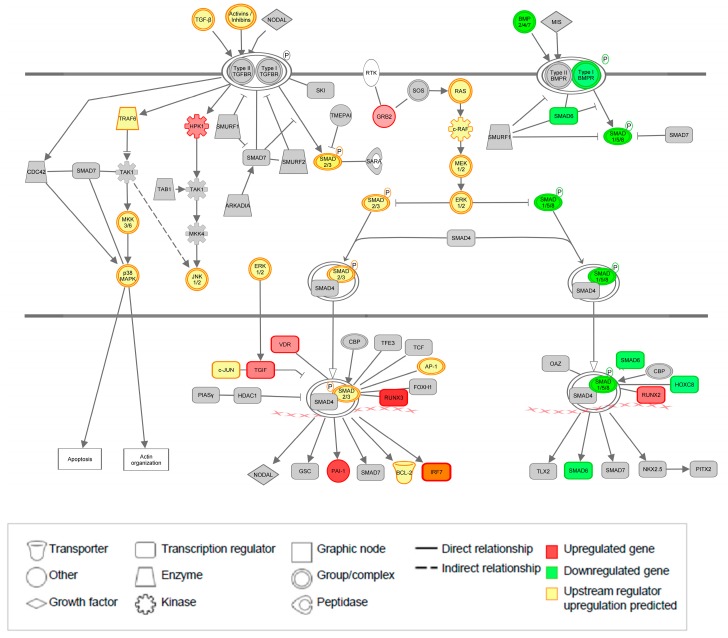
TGF-β signaling pathway with mediators in the TGF-β pathway and BMP pathway are depicted, adapted from IPA. Upregulated genes in red, down regulated genes in green, and upstream regulators which are predicted to be upregulated in yellow.

In addition to the many significantly altered inflammation pathways identified in this analysis, other interesting pathways were also significantly altered, one of which is the TGF-β signaling pathway. As the TGF-β pathway is also a determining factor in the development of TAA, we next examined this pathway more closely. Figure 5 shows the TGF-β signaling and the Bone Morphogenetic Protein (BMP)-pathway, as derived from IPA, with all genes and upstream regulators that are significantly altered. As shown, many genes and upstream regulators from our dataset are upregulated in the TGF-β pathway, e.g. the known factors TGF-β, ERK1/2, SMAD2/SMAD3, and Pai-1. Notably, IRF7 is not only upregulated at the mRNA level but also predicted to be upregulated at the upstream regulator level. Interestingly, many genes in the BMP signaling pathway were significantly downregulated, e.g. the BMP2/4/7 cytokines, Type I BMP receptor as well as the smad1/5/8 complex, which implies that this part of the pathway is inhibited in AAA disease compared to AOD. Moreover, genes involved in the pERK pathway, which regulates TGF-β as well as the BMP signaling has been previously associated with (thoracic) aneurysmal disease, are predicted to be upregulated.

## 4. Discussion

In this study we investigated the genetic factors and molecular processes that differentiate abdominal aortic aneurysm formation from arterial occlusive disease, two different clinical entities in patients with similar atherosclerotic burden. Through analysis of the differential gene expression profile we show important pathway differences, in particular in the upregulation of distinct inflammation pathways, but also differences in two previously identified TAA-related pathways; TGF-β and BMP signaling.

Clinical characteristics of the 19 patients included in our microarray dataset were analyzed and we observed no differences in the cardiovascular comorbidities and risk factors, indicating that indeed these factors do not explain the observed phenotypic differences between AAA and AOD. Similarly, a previously described patient cohort study of 1393 patients also showed no differences in cardiovascular comorbidities and risk factors, strengthening our findings [20]. Smoking, gender, obesity, age, hypertension, and dyslipidemia are associated with an increased risk for AAA, whereas diabetes, is associated with a reduced risk [52,53,54]. Indeed, we observed that diabetes is significantly lower in the AAA compared to the AOD group.

In both the patient cohort used for the micro-array RNA expression (Table 2) and the patient cohort population study we show a gender and age difference between AAA and occlusive disease patients with an overrepresentation of males in the AAA group as compared to the occlusive disease group (85.5% versus 66.9%, *p* < 0.001) [20]. This observation has also been described earlier as it is known that the incidence of AAA rises rapidly after the age of 55 years in men [10,13,15]. Therefore, our data reflect the actual AAA and occlusive disease patient population. We used a dataset of gender specific genes to correct for gender differences, as this could be an influencing factor for several upregulated genes. However, comparison of the gender-dependent and gender-independent datasets revealed only minor differences. We performed an IPA core analysis on the dataset with and without these gender specific genes and both analyses showed very similar results regarding functions, pathways, and upstream regulators, suggesting that the differences between AAA and AOD are the predominant determinants. To select AAA-specific genes irrespective of gender, we used the list of gender-independent significantly regulated genes, for further IPA analysis of AAA disease (Table 3).

From the list with significantly upregulated genes we selected a top 10 of potential markers, based on their expression level, significance, and presence in vascular tissue, and performed literature research to identify possible connections of these genes to AAA or AOD. Of these 10 genes, four showed an association with aneurysmal disease, showing that our selection procedure indeed can reveal aneurysm relevant markers. At the same time, the other six genes showed no previously known association, making them potential novel markers for AAA disease. We performed an additional qPCR validation analysis that confirmed increased expression of these five genes, showing similar upregulation in AAA samples compared to AOD. Further verification of potential AAA markers upregulation at the transcriptional level should be performed in the blood of AAA and control patients, for which an independent AAA patient cohort is needed.

The IPA analysis showed an overrepresentation of significantly up- and downregulated immune-specific pathways for AAA disease (Table 5). Moreover, analysis of hs-CRP levels in an additional patient cohort of 1393 patients showed slightly increased hs-CRP levels in AAA compared to occlusive disease patients. Although in this larger cohort we show increased inflammation based on hs-CRP, data of other known inflammation markers were not available. However, the significance of increased hs-CRP in already established aneurysms is unknown, as inflammation is a multifactorial process. Similar to what we find, other studies reported the role of the immune-related genes and pathways in AOD and AAA disease [55,56,57,58]. In the present study, however, we directly compared AAA and AOD instead of comparing both to a control group, which eliminates all genes that they have in common, or are similarly up- or downregulated. This approach is based on the fact that both diseases share common risk factors including a common atherosclerotic burden, yet present with quite different disease outcome. Therefore, the fact that we still find an overrepresentation of inflammatory pathways that differ between these two diseases, rather than being in common, is novel and should be further explored. Considering that inflammation plays an important role in both diseases [59], it is of great interest to find these many differences, indicating that the immune system as a key process in differentiating these two diseases. Therefore, the immune system and its associated markers should be further investigated in these patient groups. However, our analysis was not sufficient to pinpoint one inflammation pathway which exclusively differentiates AAA disease from AOD. More likely, we need to look for a combination of different significantly altered immune pathways, together providing an ‘immune signature’ that is different for AAA compared to AOD. This could further be explored in the blood of AAA and AOD patients. Together the changes in distinct inflammation pathways derived from our gene expression analysis, as well as the finding that hs-CRP levels that differ significantly between aortic aneurysm and occlusive disease patients, imply that a more thorough analysis of immune factors in the blood for these two patient groups is warranted.

Dysregulation of the TGF-β and BMP signaling pathway, previously described for TAA patients [8,60,61,62], was also shown for AAA patients in our IPA analysis. While we found most components of the TGF-β signaling pathway significantly upregulated, most components of the BMP-pathway were downregulated in AAA compared to AOD. Moreover, many upstream regulators involved in the TGF-β pathway were predicted to be upregulated in our analysis. Interestingly, TGF-β signaling was mostly reported to be upregulated in TAA, and intervention therapy aimed at diminishing TGF-β was able to reduce aneurysmal growth. In addition, blockade of TGF-β-signaling by TGF-β-neutralizing antibody (Nab) showed beneficial effects in MFS rodent models. In contrast, TGF-β-Nab administration exacerbated the pathology of aneurysms in angiotensin-II induced AAA mouse models [63,64]. Consequently, in AAA (dys)regulation of the TGF-β signaling pathway is not clear yet [65]. For example, a small study in 12 AAA and six control biopsies showed downregulation of TβRII subtype mRNA [66]. Yet, about 20–30% of AAA patients later in life also develop a TAA [67,68]. For example, Karkhanis et al. showed that about 16% of AAA patients studied showed major thoracic findings [69]. Vice versa, many TAA patients have aneurysms at multiple sites, including the abdominal part [70,71]. Therefore, similar mechanisms might be at work in both AAA and TAA patient groups. In this respect it is very interesting that our data show that the TGF-β signaling pathway might be dysregulated in AAA aorta samples, with predictions that the pathway is upregulated. At the same time, the closely associated BMP pathway is predicted to be downregulated. These data could indicate that an imbalance between TGF-β and BMP signaling causes part of the AAA phenotype. Several papers suggested that TGF-β actually inhibits AAA formation [72,73,74]. Although this seems contradictory to our findings, we do not necessarily show activation of the TGF-β pathway, but instead a dysregulation, which might also explain the different findings described above on TGF-β signaling pathway involvement in AAA versus TAA. It would therefore be interesting to further investigate factors involved in both the TGF-β and BMP signaling pathways in tissue or serum samples from AAA patients. In particular, measuring the TGF-β ligands 1–3 in the serum could be of great importance, in parallel to measurements of TGF-βR subtype mRNA levels. In addition, gene expression differences will also be influenced by fundamental differences in cell content and cell subtypes between AAA and AOD. In this regard, marked TGFβ and BMP signaling in AAA could reflect the trans mural fibrotic phenotype of AAA disease.

In conclusion, our data show that gene expression profiling could be an important tool to molecularly distinguish AAA from AOD, clinical entities that share the same risk factors, but show completely different disease progression, as we revealed that simultaneous inhibition of BMP signaling and activation of TGF-β signaling could play a role in abdominal aortic aneurysms. In addition, these profiles are important in the identification of novel genes, markers, and processes that can shed light on the molecular mechanisms underlying abdominal aneurysm formation.

## Figures and Tables

**Table 1:** Primer sequences used for qPCR analysis.

	Fw seq	Rev seq
**β-Actin**	CTCCCTGGAGAAGAGCTACG	GAAGGAAGGCTGGAAGAGTG
**Hprt**	TGACACTGGCAAAACAATGCA	GGTCCTTTTCACCAGCAAGCT
**COL11a1**	ACAATAGCACAGACGGAGGC	GGATTTGGCTCATTTGTCCCAG
**AdipoQ**	GTGATGGCAGAGATGGCACC	ACTCCGGTTTCACCGATGTC
**Cxcl13**	CGAATTCAAATCTTGCCCCGT	ACTTGTTCTTCTTCCAGACTATGA
**Slca5**	TCATCATCCGGCCTTCATCG	AGCAGCAGCACGCAGAG
**Fdc-sp**	GGCTGTTGGTTTCCCAGTCTC	TGGTGGAAGTGGGCGAAATG

Gene expression was calculated using actin-β and Hprt as housekeeping genes and the comparative Ct method (ΔΔCt) was used for relative quantification of gene expression.

**Table 2:** **Characteristics of the cohort used for tissue collection.** A *t*-test (continuous data) or Fisher’s exact test (categorical data) was applied for the analysis between groups. All statistical analyses were performed using Graphpad Software (Graphpad Software Inc., La Jolla, CA, USA). All statistical tests were two-sided and *p* < 0.05 was considered statistically significant.

Characteristic	AAA (n = 12)	AOD (n = 7)	*p*-Value
Male gender—n (%)	11 (92)	2 (29)	0.0095
Age—(y, mean ± SD)	68 ± 6.7	56 ± 5.7	0.001
Diabetes mellitus—n (%)	0 (0)	1 (14)	0.3684
Ischemic heart disease—n (%)	2 (17)	1 (14)	1
Renal insufficiency—n (%)	4 (33)	1 (14)	0.6027
Hypertension—n (%)	9 (75)	5 (71)	1
Dyslipidemia—n (%)	9 (75)	6 (86)	1
Current smoking—n (%)	6 (50)	4 (57)	1
Ever smoking—n (%)	4 (33)	3 (43)	1

**Table 3:** Top 30 upregulated genes (AAA gene list, part 1) in AAA versus AOD, including fold change (FC), *p*-value, location in the cell (location), type of molecule, and presence in the vascular gene set.

Gene Symbol	Entrez Gene Name	Fold Change	*p*-Value	Location	Type(s)	Vascular Gene Set
CXCL13	chemokine (C-X-C motif) ligand 13	32.269	1.12 × 10^−4^	Extracellular Space	Cytokine	YES
COL11A1	collagen, type XI, alpha 1	27.046	5.29 × 10^−5^	Extracellular Space	Other	YES
SAA2	serum amyloid A2	24.96	3.95 × 10^−7^	Extracellular Space	Other	NO
ADIPOQ	adiponectin, C1Q and collagen domain containing	21.454	3.01 × 10^−4^	Extracellular Space	Other	YES
FDCSP	follicular dendritic cell secreted protein	21.38	1.01 × 10^−4^	Extracellular Space	Other	NO
POU2AF1	POU class 2 associating factor 1	18.842	4.79 × 10^−4^	Nucleus	transcription regulator	NO
MS4A1	membrane-spanning 4-domains, subfamily A, member 1	18.414	2.61 × 10^−4^	Plasma Membrane	Other	YES
MZB1	marginal zone B and B1 cell-specific protein	17.072	1.26 × 10^−3^	Extracellular Space	Other	NO
SLC7A5	solute carrier family 7 (amino acid transporter light chain, L system), member 5	15.716	5.37 × 10^−7^	Plasma Membrane	Transporter	YES
LEP	leptin	14.288	1.94 × 10^−6^	Extracellular Space	growth factor	YES
MARCO	macrophage receptor with collagenous structure	13.563	4.12 × 10^−4^	Plasma Membrane	transmembrane receptor	YES
LPL	lipoprotein lipase	12.984	3.51 × 10^−5^	Cytoplasm	Enzyme	YES
IL1RN	interleukin 1 receptor antagonist	12.873	1.16 × 10^−3^	Extracellular Space	Cytokine	YES
IGLL5	immunoglobulin lambda-like polypeptide 1	12.848	9.73 × 10^−4^	Plasma Membrane	Other	NO
CR2	complement component (3d/Epstein Barr virus) receptor 2	12.123	1.13 × 10^−3^	Plasma Membrane	transmembrane receptor	NO
KIAA1199	KIAA1199	12.122	1.60 × 10^−3^	Cytoplasm	Other	YES
TREM1	triggering receptor expressed on myeloid cells 1	11.851	4.33 × 10^−4^	Plasma Membrane	transmembrane receptor	YES
P2RX5	purinergic receptor P2X, ligand-gated ion channel, 5	11.706	8.53 × 10^-5^	Plasma Membrane	ion channel	NO
HMOX1	heme oxygenase (decycling) 1	10.932	5.47 × 10^−5^	Cytoplasm	Enzyme	YES
IGLJ3	immunoglobulin lambda joining 3	10.776	7.29 × 10^−3^	Other	Other	NO
IGH	immunoglobulin heavy locus	10.257	4.24 × 10^−4^	Other	Other	NO
ISG20	interferon stimulated exonuclease gene 20kDa	10.238	1.31 × 10^−5^	Nucleus	Enzyme	NO
CCL18	chemokine (C-C motif) ligand 18 (pulmonary and activation-regulated)	10.164	1.51 × 10^−4^	Extracellular Space	Cytokine	YES
CD79A	CD79a molecule, immunoglobulin-associated alpha	10.064	1.97 × 10^−4^	Plasma Membrane	transmembrane receptor	NO
FNDC1	fibronectin type III domain containing 1	10.028	2.80 × 10^−4^	Plasma Membrane	Other	YES
TIMD4	T-cell immunoglobulin and mucin domain containing 4	9.859	2.59 × 10^−3^	Plasma Membrane	Other	NO
PIM2	pim-2 oncogene	9.838	2.35 × 10^−4^	Other	Kinase	YES
CXCL5	chemokine (C-X-C motif) ligand 5	9.659	2.76 × 10^−4^	Extracellular Space	Cytokine	YES
FCRL5	Fc receptor-like 5	9.596	2.49 × 10^−3^	Other	Other	NO
CXCL3	chemokine (C-X-C motif) ligand 3	9.526	3.06 × 10^−6^	Extracellular Space	Cytokine	YES

**Table 4:** Top 10 genes upregulated genes in AAA versus AOD, including their function, fold change (FC), *p*-value in dataset, location, and presence or absence in the vascular gene set.

Gene	Function and Relation to AAA or Atherosclerosis	FC	*p*-Value	Location	Vascular Gene Set
**CXCL13** C-X-C motif chemokine 13	Selective chemotactic for B cells (B-1 and B-2 subsets), by interacting with chemokine receptor CXCR5. Control of B cell organization within follicles of lymphoid tissues [25,26,27]. High levels of CXCL13 are found in aneurysm and in atherosclerotic lesions	32.26	1.12 × 10^−4^	Extracellular space	YES
**COL11A1** Collagen alpha-1(XI) chain	Adds structure and strength to connective tissues supporting muscles, joints, organs, and skin. Col11a1 protein levels are upregulated in TAA and AAA tissue [23,28,29].	27.05	5.29 × 10^−5^	Extracellular space	YES
**SAA2** Serum amyloid A protein	Production primarily in liver, circulates in low levels in the blood. Although its function is not fully understood, serum amyloid A appears to play a role in the immune system. Different biomarker studies have shown association of SAA with atherosclerotic disease. Patients with atherosclerotic disease show increased levels of Amyloid A protein [30,31,32,33].	24.96	3.95 × 10^−7^	Extracellular space	NO
**ADIPOQ** Adiponectin	Involved in the control of fat metabolism and insulin sensitivity, with direct anti-diabetic, anti-atherogenic and anti-inflammatory activities. Stimulates AMPK phosphorylation and activation in liver and skeletal muscle, enhancing glucose utilization and fatty-acid combustion. Negatively regulates TNF-alpha expression in various tissues such as liver and macrophages. Inhibits endothelial NFκB signaling through a cAMP-dependent pathway. Adiponectin is dysregulated in aneurysm and atherosclerotic disease [34,35,36].	21.45	3.01 × 10^−4^	Extracellular space	YES
**FDCSP** follicular dendritic cell-secreted protein	FDCSP bind to the surface of B-lymphoma cells. Functions as a secreted mediator acting upon B-cells. No direct associations of FDCSP with atherosclerotic disease or AAA are described in literature [37,38].	21.38	1.01 × 10^−4^	Extracellular space	NO
**POU2AF1** POU domain class 2-associating factor 1	Transcriptional coactivator that specifically associates with either OCT1 or OCT2. It boosts the OCT1 mediated promoter activity and to a lesser extent that of OCT2. Essential for the response of B-cells to antigens and required for the formation of germinal centers. Little is known about this factor in AAA, though in carotid plaque formation analysis it is shown that POU2AF1 is upregulated, which is related to the immune and inflammatory processes linked to atherosclerosis [39,40].	18.84	4.79 ×10^−4^	Nucleus	NO
**MS4A1** membrane-spanning 4A/CD20	B-lymphocyte surface molecule which plays a role in the development and differentiation of B-cells into plasma cells. B-lymphocytes with MS4A1 expressed are found in aneurysm and atherosclerotic [41,42,43,44].	18.41	2.61 × 10^−4^	Plasma membrane	YES
**MZB1** Marginal zone B and B1 cell-specific protein	Associates with immunoglobulin M (IgM) heavy and light chains and promotes IgM assembly and secretion. Acts as a hormone-regulated adipokine/ proinflammatory cytokine implicated in causing chronic inflammation, affecting cellular expansion and blunting insulin response in adipocytes. No direct association of MZB1 with atherosclerotic disease or AAA are described in literature [45].	17.07	1.26 × 10^−3^	Extracellular space	NO
**SLC7A5** Solute carrier family 7 member 5	Encodes for a protein called y+L amino acid transporter 1 (y+LAT-1). Involved in transport of amino acids, namely lysine, arginine, and ornithine. The y+LAT-1 protein forms one part (the light subunit) of a complex called the heterodimeric cationic amino acid transporter, responsible for binding to the amino acids that are transported. There is no direct association of SLC7A5 with atherosclerotic disease or AAA described in the literature [46].	15.72	5.37 × 10^−7^	Plasma membrane	YES
**LEP** Leptin	Hormone involved in the regulation of body weight. As fat accumulates in cells, more leptin is produced, indicating that fat stores are increasing. Increased leptin levels are associated with atherosclerotic disease. Furthermore, in an AAA animal model mRNA and protein levels of leptin were found to be upregulated in aneurysmatic tissue [47,48,49,50,51].	14.29	1.94 × 10^−6^	Extracellular space	YES

**Table 5:** List of top 30 upstream regulators (AAA gene list, part 2), predicted to be significantly different between AAA and AOD (z-score => 2.0; *p* < 0.01). FC; fold change of these genes in the data set. Target molecules from the data set are depicted on which upstream regulator prediction is based.

Upstream Regulator	Activation *z*-Score	*p*-Value of overlap	FC	Molecule Type	Predicted Activation State	Target Molecules in Dataset
**IL1B**	7.3	9 × 10^−27^	6.7	cytokine	Activated	ABCG2,ACTA2,ADAM8,ADM,AIF1,AMPD3,ANGPT1,ANGPTL4,APOB,APOE,ARC,ARG1,BCL2A1,BCL3,BGN,BIRC3,BMP4,CCL3,CCL5,CCR1,CCR5,CCR6,CCR7,CCRL2,CD14,CD4,CD44,CD83,CD86,CEBPB,CEBPD,CFLAR,CHI3L1,COL10A1,CREM,CSF2RB,CSF3,CTSB,CTSS,CTSZ,CX3CL1,CXCL1,CXCL2,CXCL3,CXCL5,CXCR4,CYBA,CYBB,CYSLTR1,CYTIP,DAB2,DDIT4,DUSP5,EDN1,ENPP1,ERBB2,ESR1,F2RL1,FABP5,FAM129A,FCGR2B,FGF2,FOSL1,FST,G0S2,GAD1,GADD45B,GBP1,GCH1,GLA,GM2A,HAS1,HEY2,HGF,HIF1A,HK2,HMGA1,HMOX1,HSD11B1,IBSP,ICAM1,IER3,IGFBP5,IGFBP6,IL10,IL10RA,IL16,IL18,IL18R1,IL18RAP,IL1B,IL1R2,IL1RN,IL33,IL6,IL6R,IL8,IRAK1,IRF1,IRF7,ISG20,ITGAM,LCP1,LEP,LIF,MCL1,MMP1,MMP12,MMP3,MMP9,MYEF2,MYH11,NAMPT,NFIL3,NR4A3,OCLN,OLR1,OSM,PCDH7,PDE4B,PIM1,PLAT,PLAU,PRKCD,PTGS1,PTGS2,PTP4A1,PTX3,RAC2,REL,RUNX2,S100A8,S100A9,SAA2,SCUBE2,SDC1,SERPINB9,SERPINE1,SLAMF1,SLC12A1,SLC14A1,SLC1A3,SLC20A1,SOD2,SPP1,SRGN,STAT4,STMN2,TAC1,TACR1,THBS1,THRSP,THY1,TLR2,TLR3,TLR8,TMEM176B,TNFAIP3,TNFRSF11A,TNFRSF1B,TREM1,TREM2,TYMP,UAP1,UGCG,VDR,VEGFA,XYLT1,ZC3H12A
**CEBPA**	4.9	1 × 10^−20^	3.1	transcription regulator	Activated	ACSL1,ADCY7,ADH1B,ADIPOQ,AGT,ALOX5AP,ANPEP,APOB,ARG1,ARL4C,BCL2A1,BTG1,C3AR1,CCR1,CD14,CD19,CD3G,CEBPA,CEBPB,CEBPD,CHI3L1,COL10A1,CSF1R,CSF3,CSF3R,CXCR4,DDX21,DGAT2,EFNB2,EMCN,FABP4,FASN,FCAR,FHL1,G0S2,GABPB1,GAS1,GATA6,GBP1,GCH1,GLRX,HCAR3,HGF,HMOX1,HSD11B1,ICAM1,IER3,IL10,IL1RN,IL6,IL6R,IL8,ITGAL,ITGAM,ITGAX,LCK,LEP,LPL,LST1,LTF,MALT1,MNDA,NFATC2,NFIL3,OLR1,PAX5,PCK1,PFN2,PGD,PLIN2,PPP1R3C,PTAFR,PTGS1,PTGS2,PTPN3,PTPRC,PTPRE,PTX3,RGS2,RUNX2,RUNX3,S100A8,S100A9,SCD,SEMA3E,SERPINE1,SMPDL3A,SOD2,SPP1,TAC1,TBXAS1,THRB,TRIB1,VDR,VLDLR
**IL6**	4.6	3 × 10^−19^	4.0	cytokine	Activated	ABCA1,ABCG2,ACP5,AGT,ANPEP,APOB,APOE,ARG1,ARL4C,BATF,BCL2L11,BCL3,BGN,C5AR1,CCL5,CCR1,CCR5,CCR6,CCR7,CD14,CD163,CD209,CD36,CD48,CD53,CD68,CD79A,CD83,CD86,CDKN2B,CEBPA,CEBPB,CEBPD,CFLAR,CLU,CSF2RB,CSF3R,CXCL1,CXCL13,CXCL2,CXCL3,CXCL5,CXCR4,CYBB,CYTIP,EZH2,GADD45B,GLRX,GSTA4,GZMB,HGF,HIF1A,HLA-DQA1,HMOX1,ICAM1,ICAM3,ICOS,IGFBP5,IGFBP6,IGHM,IGJ,IL10,IL1RN,IL6,IL6R,IL7R,IL8,IRF1,IRF4,ITGAM,JAK1,JAK2,KIAA0101,KLRB1,KRT14,LEFTY2,LEP,LIF,LPL,LRG1,LRP6,LTF,LY86,MCL1,MERTK,MMP1,MMP12,MMP3,MMP9,MRVI1,MSR1,NAMPT,NCF2,PIM1,PLAT,PLAU,PRF1,PROK2,PTGS2,PTPRC,PTTG1,RAB27A,RNASE6,RRM2,S100A9,SAA2,SEMA4A,SERPINA1,SERPINE1,SGK1,SLC14A1,SLC7A7,SNX10,SOD2,SPP1,SRA1,STAT4,TAC1,TBXAS1,THBS1,THRSP,TLR1,TLR10,TLR2,TLR3,TLR8,TNFRSF11A,TNFRSF17,TNFRSF1B,TOP2A,VEGFA,VLDLR,XBP1
**IL18**	4.4	4 × 10^−11^	2.6	cytokine	Activated	ADIPOQ,CCL3,CCL5,CCR5,CCR7,CD44,CD69,CD83,CD86,CFLAR,CXCL16,CXCL3,GADD45B,GZMB,HAVCR2,ICAM1,IL10,IL12RB1,IL18,IL18R1,IL1B,IL6,IL8,INPP5D,IRF1,ITGAM,KLRC4-KLRK1/KLRK1,MMP1,MMP3,MMP9,PRF1,PTGS2,SELL,SPP1,TACR1,TXK,VEGFA
**IRF7**	4.3	2 × 10^−3^	2.6	transcription regulator	Activated	CCL5,CCRL2,CD69,CTLA4,FAM26F,GBP1,GBP5,IRF1,IRF7,IRF8,ISG20,ITGAM,ITGAX,JAK2,MCL1,MX2,NAMPT,PELI1,PLAC8,PMAIP1,S100A8,TLR8,TMBIM6,TNFAIP8,ZBP1,ZC3HAV1
**TLR2**	4.1	9 × 10^−9^	4.3	transmembrane receptor	Activated	ARG1,CCL5,CCR1,CCR5,CD69,CD86,CEBPB,CEBPD,CSF3,CXCL2,CXCL3,CYLD,GRIN2A,GZMB,HLA-DQA1,HMOX1,ICAM1,IL10,IL18,IL1B,IL1RN,IL6,IL8,IRAK1,IRF1,ITGA4,LEP,MMP1,MMP9,PTGS2,TREM1,VDR,XBP1
**CEBPB**	4.0	3 × 10^−12^	2.4	transcription regulator	Activated	ACTA2,ADIPOQ,AGT,ALDH1A1,ALOX5AP,APOB,ARG1,BCL2A1,BLNK,CCL3,CCL5,CCR5,CD14,CDKN2B,CEBPA,CEBPB,CEBPD,CIRBP,COL10A1,CSF1R,CSF3,CSF3R,CXCL2,CXCL3,CXCL5,DAB2,DGAT2,EFNB2,EMCN,FABP4,FBLN1,FCAR,FHL1,GAS1,HGF,HSD11B1,ICAM1,IER3,IGKC,IL10,IL11RA,IL1B,IL1RN,IL6,IL8,ITGAL,LCP2,LEP,LYN,MBP,MGP,MMP1,MMP3,MSR1,NFATC2,NFKBID,PCK1,PLAUR,PRKCD,PTGS1,PTGS2,RAC2,RUNX2,SAA2,SAT1,SCD,SEMA3E,SERPINA1,SERPINE1,SGK1,SPP1,TAC1,TLR8,TMEM176B,TRIB3,UPP1,VDR,VLDLR,XIST
**CCL5**	3.9	2 × 10^−8^	2.4	cytokine	Activated	C5AR1,CCL3,CCL5,CCR1,CCR5,CCRL2,CD163,CD44,CXCL2,CXCL3,EMP1,F2RL1,HMGA1,IL1B,IL6,IL8,MMP19,MMP9,NAMPT,OLR1,PLAUR,PNP,PPIF,SGK1
**OSM**	3.8	3 × 10^−13^	6.1	cytokine	Activated	ABCA1,ABCG1,ADAM17,ADH5,AMPD3,AQP9,ARG1,ARHGEF12,ARL4C,BHLHE40,BTC,CALB2,CCL5,CEBPA,CEBPD,CH25H,CHD1,CPM,CSF3,CTSL,CXADR,CXCL1,CXCL13,CXCL2,CXCL3,CXCL5,CYP4F3,DNAJC3,DSC2,ECM2,FGF2,FOXC1,GAB1,GBP1,GLUL,GRIN2A,HGF,HIF1A,HK2,HMOX1,HOXA9,HSD11B1,ICAM1,IGFBP6,IL10,IL18,IL1B,IL1R2,IL33,IL6,IL6R,IL8,IRAK1,IRF1,IRF7,ISG20,ITGAL,JAG1,LIF,LRRFIP1,MAP2,MARCKS,MICA,MLLT11,MMP1,MMP3,MMP9,MYEF2,MYH10,NAMPT,NEDD4,NELL2,NOTCH3,NUAK1,OSM,P2RY10,PDPN,PFKFB3,PLAU,PRDM1,PTP4A1,PTPN21,S100A12,S100A8,S100A9,S100P,SEL1L,SERPINA1,SERPINE1,SLC16A3,SLC16A6,SOST,STK4,STX11,TLR2,TLR3,TMBIM6,TNC,TNFRSF11A,TOP2A,TPM1,TYMP,UAP1,VDR,VEGFA,ZBTB43,ZC3HAV1
**XBP1**	3.8	5 × 10^−4^	3.3	transcription regulator	Activated	COL10A1,CXCL2,DERL1,DNAJB9,DNAJC3,EDEM1,ERO1LB,ESR1,ETS1,FASN,FKBP11,FKBP7,HMOX1,HSPA13,ICAM1,IL6,IL8,IRF4,NCF1,PDIA4,POU2AF1,PRDM1,RUNX2,SDF2L1,SEC11C,SEC24D,SEC61A1,SERPINA1,SPCS3,SRPRB,SSR4,STARD5,TXNDC11,TXNDC5
**SELPLG**	3.7	5 × 10^−7^	3.5	other	Activated	BCL2A1,CCL3,CXCL2,CXCR4,HCAR3,HCK,IL10,IL1B,IL1R2,IL8,ITGAM,PLAUR,PRKCD,SERPINB9
**STAT4**	3.6	3 × 10^−6^	3.1	transcription regulator	Activated	ACADL,ACAP1,ARMCX1,BCL2L11,BCL3,CCR5,CXCL2,CXCL3,ERO1L,FCER1G,FYB,GRTP1,IER3,IL10,IL10RA,IL12RB1,IL18R1,IL18RAP,IL6,IRF1,IRF4,ISG20,ITGA7,KDM6B,LRRFIP1,MAP3K1,MGARP,PCGF5,PDK1,PLAC8,PRDM16,RASL12,RGCC,SAT1,SELENBP1,SELPLG,SERPINE1,SLC2A3,SMPDL3A,STC2,TPD52,VEGFA,VLDLR
**CD2**	3.6	8 × 10^−5^	2.9	transmembrane receptor	Activated	CCR7,CD4,CD44,CD48,CD86,CD8A,HLA-DPA1,ICAM1,IL10,ITGAL,PTPRC,SELL,STAT4
**CD44**	3.6	2 × 10^−9^	2.5	enzyme	Activated	ADAM8,ARHGEF12,BCAM,BGN,BIRC3,CCL5,CCR5,CCR7,CD36,CD44,CD69,CD8A,CIDEC,CLEC7A,CX3CL1,CXADR,ERBB2,FASN,IL10,IL1B,IL1R2,IL1RN,IL6,ITGA4,ITGAX,LIMS2,LTBP1,MCL1,MMP12,MMP3,MMP9,NPNT,PLAU,SELL,SMAD1,SPP1,THBS1,THY1,TLR8,TNFRSF11A,TPM2,WNT2
**TYROBP**	3.4	1 × 10^−6^	2.6	transmembrane receptor	Activated	CCL3,CCR7,CD69,CD83,CD86,FCGR2B,ICAM1,IL6,IL8,ITGAX,NOD2,TYROBP
**POU2AF1**	3.3	9 × 10^−11^	18.8	transcription regulator	Activated	CCND3,CCR5,CD79A,CD79B,IDH2,IGH,IGHA1,IGHG1,IGHM,IGK,KCNN4,LCK,MS4A6A,PAX5,PRDM1,RBP1,SDS,SPIB,SPP1
**TREM1**	3.3	2 × 10^−15^	11.9	transmembrane receptor	Activated	ABL2,AREG/AREGB,ATP1B1,CCL18,CCL3,CCL5,CCRL2,CD14,CD86,CDKN2B,CEBPB,CKS2,CRTAM,CXCL1,CXCL2,CXCL3,CXCL5,DUSP14,DUSP4,EDN1,FOSL1,GADD45B,GCLM,GLA,HAS1,HS3ST3B1,IL10,IL1B,IL6,IL6R,IL8,IRF1,ITGAX,KANK1,LAMP3,LIF,LPL,LY9,MAFF,MCOLN2,MLF1IP,MMP1,MMP19,NOD2,NRIP3,PIM2,PLAC8,PLCXD1,PTGS2,RGS1,SCG5,SFMBT2,SLAMF7,SLC1A3,SPP1,TARP,THBS1,TLR2,TNFSF15
**PLAU**	3.3	7 × 10^−6^	4.2	peptidase	Activated	ABCG1,ARG1,C5AR1,CCL5,CCR5,CXCL3,HGF,ICAM1,IL1B,IL6,MMP1,MMP12,MMP9,PLAU,PLAUR,S100A8,S100A9,SERPINE1
**ETS1**	3.3	2 × 10^−15^	2.9	transcription regulator	Activated	ANPEP,ARL4C,ATP2A3,BCL11A,BMP4,CD14,CD27,CD79A,CD79B,CRTAM,CSF1R,ERBB2,ETS1,FCGR2A,FOXD1,GZMB,GZMK,HCST,HGF,HMOX1,HPSE,HSPA6,HSPB8,ICAM1,IL10,IL2RB,INSIG1,ITGB2,ITK,JAK1,KLRC4-KLRK1/KLRK1,LAIR1,LCK,LTB,MCL1,MMP1,MMP3,MMP9,MSR1,NCF1,NFIL3,NPR1,PLAU,PRF1,RUNX2,RUNX3,SELL,SERPINE1,SLAMF6,SPP1,SRGN,TBXAS1,TGFA,THY1,TRPC1,VEGFA,WAS,ZAP70,ZEB1
**NFATC2**	3.3	2 × 10^−6^	2.5	transcription regulator	Activated	ABCA1,ACP5,BATF,CD3G,CFLAR,CTLA4,CXCL3,DAB2,E2F5,EDN1,ICOS,IKZF1,IL10,IL18,IRF1,IRF4,IRF7,ISG20,MERTK,NFATC1,PELI1,PLAT,PLK2,PPP3R1,PTGS2,PTPN1,REL,RGS1,RGS2,RILPL1,TLR3,TNFSF8
**VEGFA**	3.3	5 × 10^−6^	2.1	growth factor	Activated	ACSL1,ADH5,ANPEP,BCL2A1,BTK,CCRL2,CD34,CTSB,CTSS,CXCR4,DUSP4,DUSP5,EFNB2,ETS1,FABP4,FGF2,GBP1,GRIA2,HMOX1,ICAM1,IGFBP5,IL6,IL8,INPP5D,MCL1,MEOX2,MMP1,MMP12,MMP9,NME1,OCLN,PIM1,PLAT,PLAU,PTGS1,PTGS2,RUNX2,SCO2,SERPINE1,SNCG,SOD2,THBS1,VEGFA
**MAP3K1**	3.2	6 × 10^−4^	2.0	kinase	Activated	BIRC3,CSTA,HMOX1,IL8,MMP3,PGR,PLAU,PLAUR,PTGS2,SERPINE1,THBS1,TNC,TOP2A,TPH1
**C5AR1**	3.1	8 × 10^−7^	4.8	G-protein coupled receptor	Activated	C5AR1,CD28,CD86,CSF3,CXCL2,FCER1G,FCGR2A,FCGR2B,IL1B,IL6,IL8,SERPINE1
**IL6R**	3.1	1 × 10^−8^	2.9	transmembrane receptor	Activated	CCL3,CCL5,CD36,CXCL2,CXCL3,CXCL5,ICAM1,ICAM3,IGFBP5,IL10,IL6,IL8,IRF1,MCL1,MMP3,MMP9,NAMPT,PTGS2,TAC1,TNFRSF11A,VEGFA
**IL17RA**	3.1	1 × 10^−6^	2.2	transmembrane receptor	Activated	CCR1,CSF3,CSF3R,CXCL2,CXCL3,CXCR2,IL1B,IL6,IL8,MMP3,S100A8,S100A9
**PLAUR**	3.0	1 × 10^−3^	7.1	transmembrane receptor	Activated	C5AR1,CCL5,CTSB,CXCL3,CYBB,ITGAM,MMP3,MMP9,PLAU,PLAUR
**CD14**	3.0	1 × 10^−4^	3.0	transmembrane receptor	Activated	BCL2A1,CCL3,CCL5,CXCL2,CXCR2,IL10,IL10RA,IL1B,IL6,IL8,PTGS2,TLR2,TNFAIP3
**ICAM1**	2.9	9 × 10^−6^	2.4	transmembrane receptor	Activated	ACTA2,CCL5,CD69,CD86,CXCL2,CXCL3,ICAM1,IL1B,IL6,ITGA4,ITGAL,MMP9,VEGFA
**SAMSN1**	2.8	4 × 10^−5^	4.7	other	Activated	BATF,CXCL2,DAB2,EDN1,IL18,IL6,IRF1,IRF7,ISG20,MARCO,MERTK,PELI1,PLAT,PTGS2,RGCC,RILPL1,SDC1,TLR3,TNFSF8,XBP1,ZC3H12A
**NAMPT**	2.8	2 × 10^−6^	3.5	cytokine	Activated	CXCL1,CXCL2,IL6,IL8,MMP1,MMP3,MMP9,NAMPT,NELL2,NPY1R,TMSB15A

**Table 6:** Inflammatory markers of patients with aneurysmal or arterial occlusive disease. Abbreviations: hs-CRP: High sensitive C-reactive protein. Data presented in median with inter quartile range.

Inflammatory Marker.	Number	Total	Aortic Aneurysmal Disease	Arterial Occlusive Disease	*p*-Value
**triglyceride (mmol/L** **)**	1307	1.61 [1.16–2.27]	1.58 [1.13–2.19]	1.63 [1.18–2.34]	0.053
**high-density lipoprotein (mmol/L** **)**	1314	1.20 [0.97–1.46]	1.18 [0.97–1.42]	0.97 [1.20–1.48]	0.275
**low-density lipoprotein (mmol/L)**	1297	2.72 [2.04–3.47]	2.83 [2.13–3.53]	2.59 [1.95–3.41]	0.003
**hs-CRP (mg/L) [IQR]**	919	3.42 [1.63–6.00]	4.00 [2.00–6.01]	3.00 [1.39–5.48]	0.002

## Supplementary Materials

The following are available online at https://www.mdpi.com/2308-3425/6/4/38/s1, Table S1: Top upregulated genes in AAA vs AOD with the gender specific genes indicated, Table S2: Vascular Gene set used for gene expression analysis.
