# Supplementary material for: Inflammation and TGF-β Signaling Differ between Abdominal Aneurysms and Occlusive Disease
Source: J Cardiovasc Dev Dis. 2019 Nov 1;6(4):38. doi: 10.3390/jcdd6040038 (PMC6955744; doi:10.3390/jcdd6040038)
Supplement: Supplementary file 1 [file jcdd-06-00038-s001.zip › Suppl files IJpma et al JCDD/Suppl Table I and Suppl figure legends.docx]

**Table S1.** Top upregulated genes in AAA vs AOD with the gender specific genes marked (M) that were excluded for further analysis.

| Gene Symbol | Entrez Gene Name | Fold Change | p-value | Gender specific |
| --- | --- | --- | --- | --- |
| RPS4Y1 | ribosomal protein S4, Y-linked 1 | 48,386 | 0,00238 | M |
| CXCL13 | chemokine (C-X-C motif) ligand 13 | 32,269 | 0,000112 |  |
| DDX3Y | DEAD (Asp-Glu-Ala-Asp) box helicase 3, Y-linked | 30,326 | 0,00208 | M |
| COL11A1 | collagen, type XI, alpha 1 | 27,046 | 5,29E-05 |  |
| SAA2 | serum amyloid A2 | 24,96 | 3,95E-07 |  |
| PLIN1 | perilipin 1 | 23,989 | 9,49E-05 | M |
| ADIPOQ | adiponectin, C1Q and collagen domain containing | 21,454 | 0,000301 |  |
| FDCSP | follicular dendritic cell secreted protein | 21,38 | 0,000101 |  |
| PTX3 | pentraxin 3, long | 19,063 | 1,10E-05 | M |
| POU2AF1 | POU class 2 associating factor 1 | 18,842 | 0,000479 |  |
| MS4A1 | membrane-spanning 4-domains, subfamily A, member 1 | 18,414 | 0,000261 |  |
| KDM5D | lysine (K)-specific demethylase 5D | 17,854 | 0,00275 | M |
| MZB1 | marginal zone B and B1 cell-specific protein | 17,072 | 0,00126 |  |
| SLC7A5 | solute carrier family 7 (amino acid transporter light chain, L system), member 5 | 15,716 | 5,37E-07 |  |
| LEP | leptin | 14,288 | 1,94E-06 |  |
| MARCO | macrophage receptor with collagenous structure | 13,563 | 0,000412 |  |
| LPL | lipoprotein lipase | 12,984 | 3,51E-05 |  |
| IL1RN | interleukin 1 receptor antagonist | 12,873 | 0,00116 |  |
| IGLL5 | immunoglobulin lambda-like polypeptide 1 | 12,848 | 0,000973 |  |
| CR2 | complement component (3d/Epstein Barr virus) receptor 2 | 12,123 | 0,00113 |  |
| KIAA1199 | KIAA1199 | 12,122 | 0,0016 |  |
| TREM1 | triggering receptor expressed on myeloid cells 1 | 11,851 | 0,000433 |  |
| P2RX5 | purinergic receptor P2X, ligand-gated ion channel, 5 | 11,706 | 8,53E-05 |  |
| EIF1AY | eukaryotic translation initiation factor 1A, Y-linked | 11,554 | 0,00304 | M |
| SPAG4 | sperm associated antigen 4 | 11,463 | 0,00106 | M |
| HMOX1 | heme oxygenase (decycling) 1 | 10,932 | 5,47E-05 |  |
| IGLJ3 | immunoglobulin lambda joining 3 | 10,776 | 0,00729 |  |
| IGH | immunoglobulin heavy locus | 10,257 | 0,000424 |  |
| ISG20 | interferon stimulated exonuclease gene 20kDa | 10,238 | 1,31E-05 |  |
| CCL18 | chemokine (C-C motif) ligand 18 (pulmonary and activation-regulated) | 10,164 | 0,000151 |  |
| CD79A | CD79a molecule, immunoglobulin-associated alpha | 10,064 | 0,000197 |  |
| FNDC1 | fibronectin type III domain containing 1 | 10,028 | 0,00028 |  |
| IL8 | interleukin 8 | 9,89 | 0,00235 | M |
| TIMD4 | T-cell immunoglobulin and mucin domain containing 4 | 9,859 | 0,00259 |  |
| PIM2 | pim-2 oncogene | 9,838 | 0,000235 |  |
| CXCL5 | chemokine (C-X-C motif) ligand 5 | 9,659 | 0,000276 |  |
| FCRL5 | Fc receptor-like 5 | 9,596 | 0,00249 |  |
| CXCL3 | chemokine (C-X-C motif) ligand 3 | 9,526 | 3,06E-06 |  |
| MIAT | myocardial infarction associated transcript (non-protein coding) | 9,359 | 0,00011 |  |
| GZMB | granzyme B (granzyme 2, cytotoxic T-lymphocyte-associated serine esterase 1) | 9,211 | 0,000277 |  |
| IGHM | immunoglobulin heavy constant mu | 8,93 | 0,0051 |  |
| AQP9 | aquaporin 9 | 8,908 | 0,00445 |  |
| COMP | cartilage oligomeric matrix protein | 8,739 | 0,00511 |  |
| CXCL1 | chemokine (C-X-C motif) ligand 1 (melanoma growth stimulating activity, alpha) | 8,677 | 1,07E-05 | M |
| PAX5 | paired box 5 | 8,41 | 0,000776 |  |
| IGK | immunoglobulin kappa locus | 8,403 | 0,00257 |  |
| USP9Y | ubiquitin specific peptidase 9, Y-linked | 8,296 | 0,00493 | M |
| SYTL1 | synaptotagmin-like 1 | 8,235 | 4,38E-06 |  |
| C15orf48 | chromosome 15 open reading frame 48 | 8,225 | 0,0064 |  |
| DPH1 | diphthamide biosynthesis 1 | 8,218 | 0,00019 |  |

**Table S2.** The Vascular Gene Set consists of 4209 genes, implicated to have a role in vascular tissue. The Vascular Gene Set is constructed from HGMD, OMIM, relevant GO terms, relevant KEGG pathways, relevant Ingenuity IPA pathways, GWAS studies and the literature. Query definitions are listed in second tab (see submitted Excel File ‘Supp Table II’).
